# Sizing up the crowd: Assessing spatial integration difficulties in body size judgements across eating disorder symptomatology

**DOI:** 10.3389/fpsyg.2022.1003250

**Published:** 2023-01-06

**Authors:** Georgia Turnbull, Sophia Lego, Briana L. Kennedy, Joanna Alexi, Yanqi R. Li, Manja M. Engel, Georgina Mann, Donna M. Bayliss, Simon Farrell, Jason Bell

**Affiliations:** ^1^School of Psychological Science, University of Western Australia, Crawley, WA, Australia; ^2^Faculty of Social and Behavioural Sciences, Experimental Psychology, Helmholtz Institute, Utrecht University, Utrecht, Netherlands

**Keywords:** body size judgements, perceptual body image disturbance, integration, virtual reality, perception

## Abstract

**Introduction:**

Body size judgements are frequently biased, or inaccurate, and these errors are further exaggerated for individuals with eating disorders. Within the eating disorder literature, it has been suggested that exaggerated errors in body size judgements are due to difficulties with integration. Across two experiments, we developed a novel integration task, named the Ebbinghaus Illusion for Bodies in Virtual Reality (VR), to assess whether nearby bodies influence the perceived size of a single body. VR was used to simulate the appearance of a small crowd around a central target body.

**Method and Results:**

In Experiment 1 (*N* = 412), participants were required to judge the size of a central female target within a crowd. Experiment 1 revealed an Ebbinghaus Illusion, in which a central female appeared larger when surrounded by small distractors, but comparatively smaller when surrounded by large distractors. In other words, the findings of Experiment 1 demonstrate that surrounding crowd information is integrated when judging an individual’s body size; a novel measure of spatial integration (i.e., an Ebbinghaus Illusion for Bodies in VR). In Experiment 2 (*N* = 96), female participants were selected based on high (*n* = 43) and low (*n* = 53) eating disorder symptomatology. We examined whether the magnitude of this illusion would differ amongst those with elevated versus low eating disorder symptomatology, in accordance with weak central coherence theory, with the high symptomatology group displaying less spatial integration relative to the low group. The results of Experiment 2 similarly found an Ebbinghaus Illusion for Bodies in VR. However, illusion magnitude did not vary across high and low symptomatology groups.

**Discussion:**

Overall, these findings demonstrate that surrounding crowd information is integrated when judging individual body size; however, those with elevated eating disorder symptomatology did not show any integration deficit on this broader measure of spatial integration.

## 1. Introduction

Body dissatisfaction is recognized as a serious public health concern. For example, the vast majority of Australian women (up to 80%) report some level of dissatisfaction with their weight and shape (Griffiths et al., 2016, 224). This is particularly concerning given that body dissatisfaction and the overvaluation of body size, weight, and shape are recognized as core contributors to the emergence and maintenance of eating disorders (Stice and Shaw, 2002; Allen et al., 2008; Fursland et al., 2012). Eating disorders, including anorexia nervosa, bulimia nervosa, and binge eating disorder, are the third most prevalent chronic illness among adolescent girls (Hoek, 2006; Yeo and Hughes, 2011), and are associated with high levels of functional impairment, high risk for future mental and physical problems, and high mortality rates among individuals experiencing an eating disorder (Berkman et al., 2007; Steinhausen, 2009; Micali et al., 2015; Stice et al., 2017).

When it comes to perceptions of body size, research has shown that judgements are frequently biased or inaccurate. For healthy individuals, the tendency to misjudge body size occur in both the judgement of one’s own body size (Tovée et al., 2003; Wardle et al., 2006; Edwards et al., 2010; Costa et al., 2015; Thaler et al., 2018) and when judging the body size of others (Oldham and Robinson, 2016; Alexi et al., 2018; Gledhill et al., 2019). Importantly, these inaccuracies can lead to difficulties in individuals identifying weight gain or loss in themselves or others. As a result, this may hinder efforts to modify weight-related health behaviors (Solmi et al., 2020).

Moreover, these inaccuracies in body size judgements have significant clinical relevance. Errors in body size judgements have been found to occur more frequently and are more exaggerated among individuals with or at risk of an eating disorder (Whitehouse et al., 1988; Cazzato et al., 2016; Mohr et al., 2016; Moelbert et al., 2017; Gledhill et al., 2019; Alexi et al., 2019b). For example, patients diagnosed with anorexia nervosa or bulimia nervosa have been shown to overestimate their own body size (Williamson et al., 1993; Gardner and Bokenkamp, 1996; Tovée et al., 2003; Engel and Keizer, 2017; Moelbert et al., 2017) and have been shown to misjudge the body size of others (Tovée et al., 2000). Individuals with binge eating disorder have also been shown to overestimate their body size relative to peers (Nicoli and Junior, 2011).

Severe disturbances in body image can present as extreme for individuals with eating disorders. Not only do body image disturbances further motivate weight-loss behaviors, but can play a key role in the initiation, maintenance (Casper et al., 1979; Gadsby, 2017), and relapse of eating disorders (Castro et al., 2004; Keel et al., 2005; Carter et al., 2012; Gadsby, 2017), as well as being a predictor of decreased treatment success (Roy and Meilleur, 2010). Given the key role that body image disturbances play for those with eating disorders, the current study sought to further understanding of the factors that contribute to body size judgement errors and how these might vary according to eating disorder symptomatology.

Body image disturbances are theorized to be multidimensional. The general consensus is that these dimensions include cognitive/affective, behavioral, and perceptual processes (Cash and Deagle, 1997; Cash et al., 2002; Gaudio et al., 2014; Lewer et al., 2016). Regarding perceptual biases, which are the focus of this study, it has been found that past experience of body shapes can influence body size judgements in a number of ways. One well-established cause of perceptual bias is due to adaptation after-effects, where prolonged exposure to a visual stimulus (typically minutes or longer) can distort the appearance of subsequently viewed stimuli, allowing them to appear more perceptually distinct than they are (Gibson and Radner, 1937). In regards to body size judgements, it has been repeatedly demonstrated that viewing thin body shapes for a prolonged period of time causes subsequently viewed average-sized bodies to appear larger, and vice versa (Winkler and Rhodes, 2005; Hummel et al., 2012, 2013; Robinson and Kirkham, 2014; Brooks et al., 2016; Challinor et al., 2017). A second perceptual bias that tends to occur under more brief exposures is serial dependence, in which judgements are biased by and *towards* prior experience (Fischer and Whitney, 2014). Serial dependence has been observed for body size judgements, with bodies being perceived to be smaller when preceded by a smaller body, and bodies perceived as larger when preceded by a larger body (Alexi et al., 2018, 2019b; Turnbull et al., 2022). Thirdly, it has long been observed that estimates of large and small magnitudes are biased towards a mean magnitude (Hollingworth, 1910). This perceptual bias for magnitude estimation has been demonstrated in body size judgements, in which healthy participants have been shown to be relatively accurate when judging average body sizes (which tends to be between 60—70 kg). However, participants would increasingly overestimate the size of smaller/lighter bodies below this average, and increasing underestimate the size of larger bodies above this average (Cornelissen et al., 2015, 2016).

These perceptual biases constitute known sources of body size judgement errors for healthy individuals. Critically, individuals high in eating disorder symptomatology exhibit greater distortions in at least some of these perceptual biases (Cornelissen et al., 2013; Mohr et al., 2016; Gledhill et al., 2019; Alexi et al., 2019b). A theory that has gained currency for explaining why individuals high in eating disorder symptomatology may exhibit greater perceptual biases attributes those biases to deficits in integration. Integration is the process of which multiple sources of input are merged with fluency and combined to form a unified representation of an object, action, or context (Iarocci and McDonald, 2006). This concept arose from research showing that individuals with eating disorders, or high in eating disorder symptomatology have difficulties with the integration of certain information, including impaired integration of multisensory information (Eshkevari et al., 2014; Riva and Dakanalis, 2018; Riva and Gaudio, 2018) and impaired spatial integration (e.g., weak central coherence). Weak central coherence has been defined by two components: a poorer ability at global processing (forming a holistic representation) and enhanced local (feature-specific) processing abilities (Happé and Booth, 2008). It has been referred to as a “piecemeal” perceptual processing style characterized by a local bias towards specific information and failing to integrate this information into a global percept (Frith, 1989; Rouse et al., 2004; Bölte et al., 2007; Happé and Booth, 2008). According to Frith (1989), weak central coherence reflects a relative difficulty in integrating surrounding spatial information necessary to obtain higher-order meaning within the context (Frith, 1989). Past research has used different types of measures to investigate weak central coherence. These include the use of global versus local processing paradigm to illustrate deficits in global processing and strengths in local/detail processing. These types of tests include visual tasks such as Group/Embedded Figures Tests (Lang et al., 2014a,b), the Rey Osterrieth Complex Figure test (Danner et al., 2012; Keegan et al., 2021), the Fragmented Pictures Task (Harrison et al., 2011), and Object Assembly (Van Autreve et al., 2013). Additionally, research has tested weak central coherence by looking at how strongly elements/features are being integrated into one’s overall percept. This has been measured using visual tasks, including the Ebbinghaus Illusion (e.g., Happé, 1996; Ropar and Mitchell, 1999; Bölte et al., 2007; de Fockert et al., 2007; Gori et al., 2016), Ponzo Illusion (Shen et al., 2015), and the Muller-Lyer illusion (Ropar and Mitchell, 2001). Within a clinical context, difficulties with integration have been shown to occur in different populations, with weak central coherence postulated to underlie core symptomatology of autism spectrum disorders (ASD; Frith, 1989). Notably, it has been found that there is considerable comorbidity between autism and eating disorders (Berkman et al., 2007; Oldershaw et al., 2011; Huke et al., 2013). Indeed, it has also been hypothesized that autistic traits places women at a higher risk of developing anorexia nervosa (Oldershaw et al., 2011). A review by Huke et al. (2013) found that the prevalence of autism in eating disorder populations was significantly higher than that within a healthy control sample, ranging from 8 to 37%. Additionally, there is considerable overlap in traits such as weak central coherence, inflexibility, adherence to routine, restricted interests, social and flexibility difficulties, and repetitive behaviors for both individuals with autism and individuals with eating disorders (Hambrook et al., 2008; Lopez et al., 2008a,b,c; Pooni et al., 2012; Treasure, 2013; Lang et al., 2014a, 2021; Mandy and Tchanturia, 2015; Dell'Osso et al., 2018; Smith et al., 2018; Kerr-Gaffney et al., 2020; Inoue et al., 2021; Keegan et al., 2021). Thus, evidence suggests that both individuals with autism and eating disorder symptomatology share a common perceptual bias related to weak central coherence.

While a substantial body of literature acknowledges that difficulties with integration are relevant to understanding body image disturbances, the vast majority of previous research has focused on body size judgements using a single-test body stimuli (Gardner and Bokenkamp, 1996; Urgesi et al., 2012, 2014; Brooks et al., 2016; Cornelissen et al., 2016; Alexi et al., 2018, 2019b). Therefore, questions have largely been restricted to investigating the role that integration has within a single-test body stimulus, such as holistic versus local processing of bodies (Brooks et al., 2018) and multibody sensory integration (Keizer et al., 2012, 2013; Metral et al., 2014; Beckmann et al., 2021). However, we are aware of one study which has provided preliminary evidence of the influence of surrounding bodies on one’s body size judgement (Bateson et al., 2014). In this study, participants were simultaneously presented with two stimulus sets. Each stimulus set displayed a central target body, standing in between two bodies of either a lower or higher body mass index (BMI) class. Specifically, Bateson et al. (2014) found that two surrounding bodies biased the probability of a central target body being perceived as larger or smaller. A target female body was more likely to be judged thinner when standing in between two women with a higher body mass index and more likely to be judged larger when standing in between two women with a lower BMI. However, these influences were not the primary focus for Bateson et al. (2014), with the aforementioned study not measuring the *size* of this influence. In addition, Bateson et al. (2014) only used two surrounding bodies, rather than looking at integration across a crowd of body sizes. The current study directly tackles the question of whether the body size characteristics of a surrounding crowd influence the perceived size of a central target body, how that varies with body size and importantly, whether that influence differs as a function of eating disorder symptomatology. To simulate a crowd in an ecologically valid manner, body size judgements were measured within an immersive Virtual Reality (VR) setting. This 3D VR environment minimized occlusions between bodies and allowed the central target to appear distanced and distinct from the bystanders as they would in the real world.

**Figure 1:**
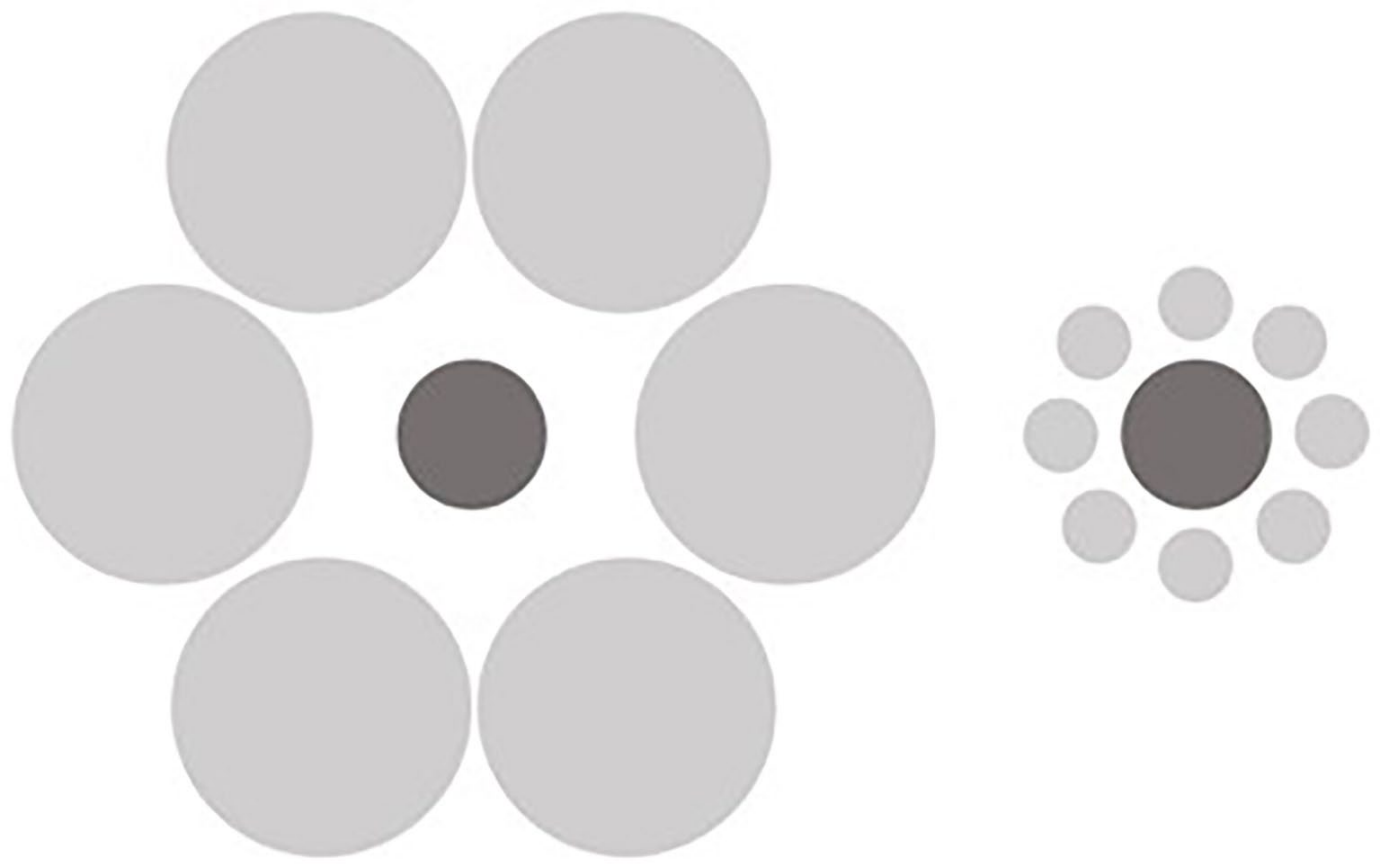
The Ebbinghaus illusion.

There is a long established paradigm for studying these types of size illusions. For instance, the Ebbinghaus Illusion (Ebbinghaus, 1902) is a well-established measure that has been used to demonstrate and study spatial integration (Happé, 1996; Gori et al., 2016). The Ebbinghaus Illusion is a visual size-contrast illusion, where the perceived size of the central target stimulus (e.g., the central circle) is altered by the surrounding stimuli (e.g., the large or small surrounding circles). As shown in Figure 1, the central circle appears larger when surrounded by smaller circles and the central circle appears smaller when surrounded by larger circles. The Ebbinghaus Illusion demonstrates how the integration of surrounding spatial information across visual scenes can bias one’s judgements of size. As evidence of its utility to studies of integration and central coherence, individuals with autism have been shown to exhibit a *smaller* Ebbinghaus Illusion, relative to individuals without autism (Happé, 1996; Bölte et al., 2007). Given the evidence that those with eating disorders similarly display weak central coherence, it seems plausible that those high in eating disorder symptomatology might similarly exhibit reduced spatial integration on these types of perceptual tasks/illusions, compared with those low in symptomatology.

The first step in such an investigation might begin by asking whether this type of size contrasting illusion occurs for body size judgements. That is, whether we integrate surrounding information (e.g., other bodies) into our judgements of body size. As discussed above, previous literature suggests that that it can (Bateson et al., 2014). We add to that preliminary finding by systematically varying the size of the target and bystanders, in order to help characterize the circumstances in which surrounding bodies do and do not influence size judgements. To simulate a crowd in an ecologically valid manner, body size judgements were measured within an immersive Virtual Reality (VR) setting. This 3D VR environment minimized occlusions and allowed the central target to appear distanced and distinct from the bystanders as they would in the real world.

Accordingly, the aim of Experiment 1 was to develop and demonstrate a novel test of spatial integration — that is, an Ebbinghaus Illusion for Bodies in VR. Specifically, we aimed to investigate if and to what extent the perceived size of a single central female body is influenced by the presence of other female bodies within the scene. We hypothesized that there would be an Ebbinghaus Illusion for Bodies in VR, that is, participants would judge the central body to be larger when surrounded by a crowd of very thin bodies (the Small Distractors) and judge the central body to be smaller when surrounded by a crowd of very overweight bodies (the Large Distractors) within a community sample. Based on findings for the traditional Ebbinghaus Illusion, we expect this bias in size judgement to increase as the difference in size of target and surrounding bodies increases (Roberts et al., 2005). In this light, we would not expect an Ebbinghaus Illusion to arise when the size of the central target body and surrounding bodies are of the same size.

Having demonstrated a significant Ebbinghaus Illusion for Body Size in a community sample, Experiment 2 then examined whether the magnitude of this illusion differed amongst those with elevated versus low eating disorder symptomatology. In accordance with weak central coherence theory, and after controlling for the contribution of traits associated with autism, it was hypothesized that individuals high in eating disorder symptomatology would display a *smaller* Ebbinghaus Illusion for Bodies in VR relative to individuals low in eating disorder symptomatology, who would likely mimic the pattern of the general population. That is, individuals high in eating disorder symptomatology are expected to display less spatial integration of surrounding bodies when judging the size of a central target body.

## 2. Experiment 1

### 2.1. Method

#### 2.1.1. Participants

The participants in this study were first year psychology students from The University of Western Australia. All students were given the option to partake in this experiment as part of their laboratory classes and informed consent was obtained. There was no compensation for participation and students were not penalized if they decided not to partake in this experiment. At the end of the experiment, students were asked if they consented to their anonymous data being used for broader research purposes. Only those students who agreed to that use of data were used in this study. Therefore, this study reports data from 419 participants. Seven participants were excluded from the study as they did not discriminate between the body sizes (i.e., they clicked the same area on the response scale throughout the task). This left us with a sample of 412 participants. Previous studies on the Ebbinghaus Illusion have tended to use relatively small samples (e.g., sample sizes of four to 36 participants in classic papers such as Ropar and Mitchell, 1999, 2001; Roberts et al., 2005), and the effect size of the Illusion is not often reported. As such, we accommodated for a small effect size by using a sample from a large undergraduate unit. Calculated with G*Power (Faul et al., 2007), a sensitivity analysis indicated that our sample size of 412 participants would allow us to observe effect sizes for our within-between subject interaction of *f* = 0.067, α = 0.05 and power (1 − β) = 0.81. The ages of the sample ranged from 18 to 52 years old (*M* = 20.20; *SD* = 5.53). The gender breakdown of the sample included 251 female participants (60.92%), 159 male participants (38.59%), and two individuals who identified as other (0.49%). The experimental procedure was approved by the University of Western Australia’s Human Research Ethics Committee and the experiment was performed in accordance with their guidelines and regulations.

#### 2.1.2. Materials

##### 2.1.2.1. Apparatus

Participants wore one of 12 head-mounted HTC Vive Pro™ devices (HTC, Valve Corporation, 2018) where each was connected to a laptop computer (15.6″ Gigabyte Sabre 15-W8, FHD, 120 Hz, Intel® UHD Graphics 630).

##### 2.1.2.2. Stimuli

**Figure 2:**
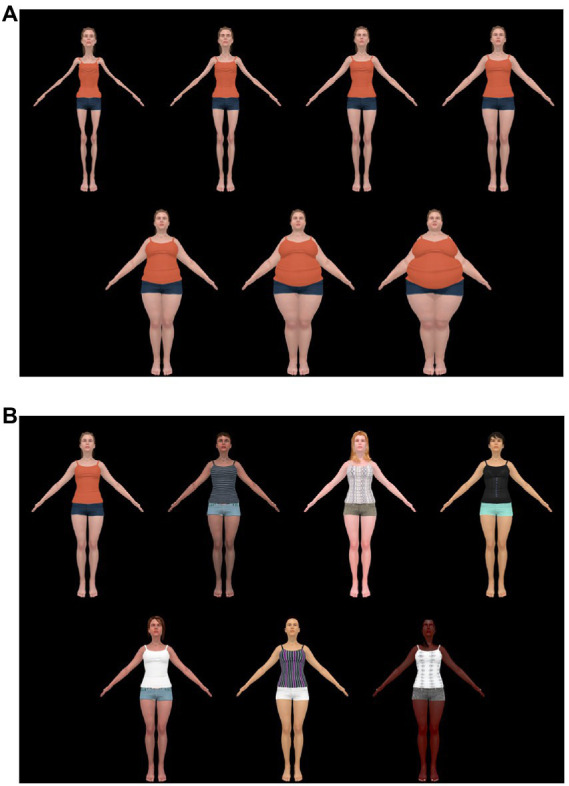
**(A)** Examples of the seven body stimuli from body Size 1 (very underweight) to 7 (very overweight). On the top row, from left to right, are bodies Size 1, Size 2, Size 3, and Size 4. On the bottom row, from left to right, are bodies Size 5, Size 6, and Size 7. **(B)** Examples of the seven body identities. The original source of these images can be found at Turnbull et al. (2022). The stimuli was created using ©Character Creator and using Shape Keys (e.g., an animation industry technique which smoothly interpolates between the vertices of the two models) to create the intermediate body types by linearly interpolating between the Size 1 and Size 4 bodies. Both figures are reproduced from Turnbull et al. (2022), with permission from SAGE Publications.

Forty-nine synthetic computer-generated images (CGI) of female bodies were created for our earlier work (Turnbull et al., 2022), ranging from very underweight (Size 1) to very overweight (Size 7). Using the program Blender 2.79© we created a linear continuum of seven discrete body size categories. Seven unique identities were created for each body size category. The Size 1, Size 4, and Size 7 body size categories were created first in Character Creator©. They were matched by the research team to be perceptually equivalent to the sizes of the smallest or largest body stimuli used in earlier work using the same task and real body images (Alexi et al., 2018, 2019b). Utilizing Shape Keys (e.g., an animation industry technique which smoothly interpolates between the vertices of the two models), we were able to generate the intermediate body types by linearly interpolating between the Size 1 and Size 4 bodies, and between the Size 4 and Size 7 bodies, in equal steps. This generated the Size 2, Size 3, Size 5, and Size 6 bodies. The bodies appeared with their arms somewhat outstretched so that arm position/horizontal extent did not need to vary with body size. In the VR environment, the bodies were 1.8 m tall and were presented approximately 2.2 m away from the participant. All bodies faced the participant. There were seven randomly generated “identities” of each body size, which varied in appearance (i.e., skin color, hair color) and in clothing (i.e., color, pattern). This was to avoid participants associating a specific identity with a specific response. Examples of the body size categories of the body stimuli are shown in Figure 2A and examples of the differing identities are shown in Figure 2B.

##### 2.1.2.3. Ebbinghaus illusion for bodies’ configuration

**Figure 3:**
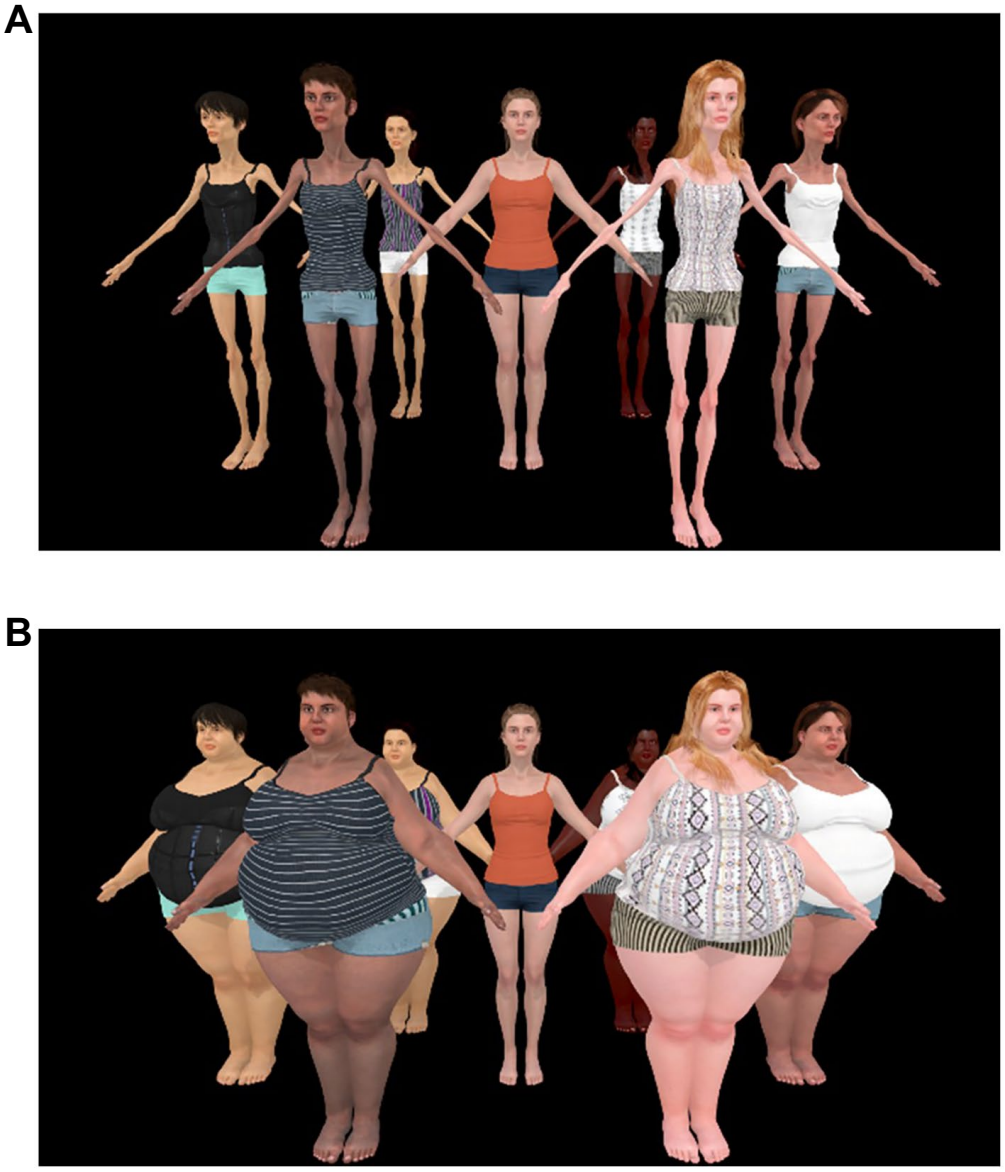
**(A)** Stimuli shown for participants in the “small distractor” condition, with the central body (Size 4) surrounded by very underweight inducers (Size 1 bodies). **(B)** Stimuli shown for participants in the “large distractor” condition, with the central body (Size 4) surrounded by six very overweight inducers (Size 7 Bodies). The stimuli was created using ©Character Creator and using Shape Keys (e.g., an animation industry technique which smoothly interpolates between the vertices of the two models) to create the intermediate body types by linearly interpolating between the Size 1 and Size 4 bodies.

As shown in Figures 3A,B, the scene presented to participants consisted of one central body (the to-be-judged body) surrounded by six bodies (inducers) to simulate a “crowd.” The central body had a red fixation cross in the center of the body, indicating that this was the body to be judged. This configuration contains broad range of locations for where the inducers located. The six inducers were positioned equidistance from each other on a 1 m radius around the central body and placed to minimize occlusion. This configuration meant that the two furthest inducers were standing 24° left and right from the central body, respectively. The four remaining inducers were standing between 10 and 14° left and right from the central body. These four closest inducers were placed in a range that is typical for the Ebbinghaus Illusion (Roberts et al., 2005). In the Small Distractor condition, the six inducers were all Size 1 bodies, as illustrated in Figure 3A. In the Large Distractor condition, the six inducers were all Size 7 bodies, as illustrated in Figure 3B.

##### 2.1.2.4. Bodyline task

Our VR bodyline task was adapted from the original *bodyline* task developed by Alexi et al. (2018) to measure body size judgements. The task was run through “Unity” software (Unity Technologies, 2019). Participants were instructed to make a body size judgement of the central female target only. To be clear, this was a judgement about *other bodies*, made from a third-person point of view, not a judgement of the body relative to the crowd, or of one’s *own* body size. This body size judgement was made using the *bodyline*, a visual analogue scale (VAS) consisting of a white, unmarked horizontal line scored from 1.0 to 7.0 (Alexi et al., 2018, 2019b). The VAS represented a continuum, in which participants were given explicit verbal instructions to make a body size judgement of the central target body. Participants were informed that the left of the VAS corresponded with the central body being judged as very underweight, and the right side corresponded with the central body being judged as very overweight. Unlike the traditional method developed and used by Alexi et al. (2018), here the VAS did not have any anchor images at either end of the scale. Each trial began with a blank black screen and a red fixation cross in the center. The crowd of bodies were then presented for 500 ms, which is double the presentation time of stimuli in previous research using the bodyline task (i.e., 250 ms; Alexi et al., 2018, 2019a,b). The longer presentation was intended to allow participants sufficient time to orient themselves to the target stimuli within the VR environment. After the presentation time, the target bodies disappeared and participants were required to respond on the VAS before proceeding to the next trial. There was a 1 s interval between trials. The VAS remained on the screen between stimuli presentations. For make their responses, participants saw a “laser beam” emitted from the controller. Responses were made by pushing down the trigger button on the HTC Vive Pro hand controller, at a location which corresponded to the participants’ judgement on the VAS.

#### 2.1.3. Procedure

This study was conducted in a series of time-limited undergraduate laboratory classes, where up to 12 participants were able to be tested at the same time. All participants gave informed consent and were fitted with the HTC Vive Pro headset and given a VR hand controller. In the VR environment, participants entered their age and gender. Participants then read a series of instructions for the bodyline task, instructing them to judge the size of the central body stimulus and to ignore the surrounding inducers.

Participants within each class were randomly assigned to one of two conditions; the Small Distractors condition, or the Large Distractors condition. In each condition participants completed 14 practice trials, in which they were presented with the full spectrum of central target body stimuli sizes (Size 1–7) twice. Following the practice trials, participants completed 150 trials. Consistent with prior research, the presentation order of the body images was fixed across all participants to ensure that each body size category both preceded and followed each other Body Size category, including its own, an equivalent number of times (Alexi et al., 2018, 2019b). This order is important for assessing serial dependencies (trial to trial perceptual biases), which are not assessed in the current study, but we replicated the original task for consistency with previous research. The experiment took approximately 15 min to complete for each participant. Following the completion of their task, participants were debriefed on the goals of the experiment.

### 2.2. Results

#### 2.2.1. Data screening

Prior to data analyses, all data for each participant were screened. Each individual’s body size judgement data were fit with a linear regression. If the fitted slope was not statistically greater than zero, it indicated that the individual did not perceive any differences between the seven body size categories and resulted in their subsequent removal from analyses. As previously mentioned, this resulted in the removal of seven participant’s data, as they did not discriminate between the seven body size categories.

Data were also assessed for outliers. Individual scores were examined according to the outlier criterion of three standard deviations above or below the mean for that variable (Howell et al., 1998; Alexi et al., 2019a,b). All outliers that were identified were subsequently Winsorized (Reifman and Keyton, 2010). Next, normality was assessed using the criterion of skew <|2.00| and kurtosis <|7.00| (Curran et al., 1996; Gignac, 2019). All variables achieved sufficient normality, with skew and kurtosis values falling within accepted limits.

#### 2.2.2. Assessment of participant differences between conditions

To verify that the Small Distractor and Large Distractor groups did not significantly differ on any baseline variables, an independent samples *t*-test was conducted to compare age. No significant difference was found between the Small Distractor and Large Distractor conditions for age, *t*(410) = 0.832, *p* = 0.406. In addition, a chi-square analysis was conducted to compare gender between the Small and Large Distractor conditions. There were no significant differences in breakdown of gender, χ^2^(2) = 0.960, *p* = 0.619. Descriptive characteristics for these variables are displayed in Table 1.

**Table 1:** Means (SD) for age and gender breakdown for the small and large distractor conditions.

Variable	Small distractors (*N* = 212)	Large distractors (*N* = 200)
Age	19.71 (3.66)	20.04 (4.43)
Gender	Female = 134 Male = 77 Other = 1	Female = 117 Male = 82 Other = 1

#### 2.2.3. An Ebbinghaus illusion for bodies

**Figure 4:**
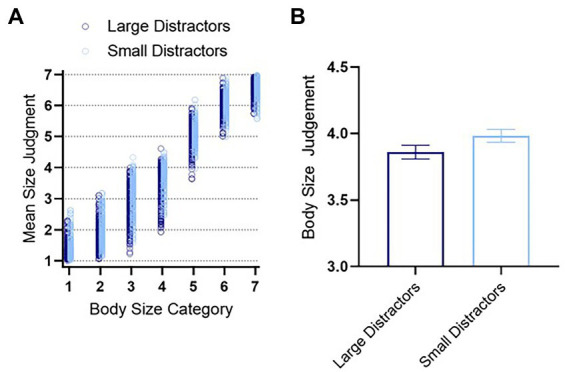
**(A)** Mean individual ratings for each body size category for all participants. **(B)** A summary of mean body size judgements for the small distractor and large distractor conditions. There was a significant difference between the conditions. Error bars represent ± 1 standard error of the mean. In **(A)**, the data for the small distractor condition has been nudged horizontally, for clarity. Created using GraphPad Prism.

Each participant’s mean size judgement in each condition is displayed in Figure 4A. To address our primary research aim, determining whether there is an Ebbinghaus Illusion when making body size judgements, we conducted a 2 (Distractor condition: Small Distractors, Large Distractors) × 7 (body size category: 1–7) mixed-model ANOVA, with body size judgements as the dependent variable.^1^

All statistical assumptions were examined. The assumption of sphericity was tested using Mauchly’s Test of Sphericity. This assumption was violated for body size category. As such, the conservative Greenhouse–Geisser adjustment was used (Gignac, 2019). All other statistical assumptions were satisfied.

As expected, there was a large significant main effect of body size category, *F*(2.59, 1064.15) = 12539.72, *p* < 0.001, partial η^2^ = 0.968, demonstrating that participants were able to discriminate between the seven body size categories, judging the central body to be larger when it was physically larger.

Next, there was a small significant main effect of distractor condition on body size judgements, *F*(1, 410) = 20.954, *p* < 0.001, partial η^2^ = 0.049. On average, bodies were judged to be *larger* when surrounded by the Small Distractors and judged to be *smaller* when surrounded by Large Distractors (Figure 4A). This is consistent with the traditional Ebbinghaus Illusion and therefore, our results demonstrate an Ebbinghaus Illusion for Bodies in VR.

The above described main effect is somewhat qualified by the presence of a small significant interaction between distractor condition and body size category, *F*(2.59, 1063.15) = 8.37, *p* < 0.001, partial η^2^ = 0.020. However, inspection of the data for each body size category in Figure 4A indicates that this interaction simply reflects variation in the magnitude of the Ebbinghaus Illusion for Bodies across body size category. That is, six out of seven body size categories appear to show the typical Ebbinghaus Illusion, such that the central target body was judged to be *larger* when surrounded by Small Distractors and judged to be *smaller* when surrounded by Large Distractors. The exception was the largest body category (Size 7), where judgements for the Small Distractor and Large Distractor conditions were equivalent. To formalize that interpretation, we used Bonferroni’s post-hoc analyses, corrected for multiple comparisons. This analysis revealed that the Ebbinghaus Illusion for Bodies in VR was significant for 3 out of the 7 body size categories (body size category 2–4). The non-significant Ebbinghaus Illusion for larger body sizes (5–7) is not surprising given participant reports of poorer precision in judging larger bodies and given the additional occlusions created in the Large Distractor condition. These points will be discussed in further detail in the General Discussion. In short, our experiment was designed to assess the nature of the surrounding bodies’ influence for a range of body sizes. However, if we simply collapse across body size to restrict the question to whether or not perceived size varies by distractor condition (i.e., an Ebbinghaus Illusion), then the answer is yes, there is a small significant effect *t*(2882) = 1.73, *p* = 0.042, *d* = 0.064, as shown in Figure 4B.

### 2.3. Discussion

The results of Experiment 1 demonstrate an Ebbinghaus Illusion for Bodies in VR, indicating that spatial integration of crowd information can lead to biased perceptual judgements of individual body size. It was found that the central body was generally perceived to be *larger* when surrounded by Small Distractors and perceived to be *smaller* when surrounded by Large Distractors. There was variation in the magnitude of the Ebbinghaus Illusion for Bodies, reinforcing the importance of measuring the illusion across a range of body sizes. Essentially, the illusion was greatest when the target body is average sized (2–4), and therefore maximally different from both the Small (Size 1) and Large (Size 7) distractors. Based on findings for the traditional Ebbinghaus Illusion (Roberts et al., 2005), we expected this bias in size judgement to increase as the difference in size of the central target and the surrounding bodies increased. These findings then add to the existing literature on the factors that influence body size judgements and associated. Specifically, we build on Bateson et al. (2014), by demonstrating that the influence of the crowd on judgements of perceived body size depends on the size relationship between the target and the bystander (inducer) and demonstrate the utility of VR technology that mimics real world situations for studying body size judgements.

## 3. Experiment 2

Experiment 1 confirmed our novel Ebbinghaus Illusion for Bodies in VR to be a valid measure of spatial integration of bodies. The aim of Experiment 2 was to investigate whether the magnitude of Ebbinghaus Illusion for Bodies varies amongst individuals differing in eating disorder symptomatology. Based on prior reports of weak spatial integration by those high in eating disorder symptomatology (e.g., Lopez et al., 2008a,b,c; Lang et al., 2014a) we hypothesized that those high in eating disorder symptoms would exhibit a smaller Ebbinghaus Illusion for Bodies than those low in eating disorder symptoms.

### 3.1. Method

#### 3.1.1. Participants

A total of 96 first year undergraduate psychology students from the University of Western Australia were invited to participate in return for course credit. Only female students aged 17—25 were eligible to participate, as research indicates this demographic to be most at risk in the development of eating disorders (Hudson et al., 2007; Stice et al., 2013; Rohde et al., 2017) and is consistent with previous related studies (Dondzilo et al., 2017; Alexi et al., 2019b). Calculated with G*Power (Faul et al., 2007), this sample size afforded us the ability to observe an Ebbinghaus Illusion for Bodies for our within-subject interaction with effects of *f* = 0.15, α = 0.05 and power (1 − β) = 0.81, and to observe effect sizes as small as *f* = 0.291, α = 0.05 and power (1 − β) = 0.81 for the comparison of the Ebbinghaus Illusion between eating disorder groups. The age of the sample ranged from 17 to 24 years old (*M* = 18.58; *SD* = 1.38), with a mean BMI of 21.56 (*SD* = 4.06), falling within the healthy BMI weight range (Flegal et al., 2014). All participants reported normal or corrected to normal (e.g., contact lenses or glasses) visual acuity. Participants were able to wear visual corrective aids, if required, within the Virtual Reality headsets. The experimental procedure was approved by the institutional Human Research Ethics Committee and the experiment was performed in accordance with their guidelines and regulations.

#### 3.1.2. Materials

##### 3.1.2.1. Titmus test

The Titmus stereoacuity test (Stereo Optical, 2017) was administered as a pre-screening measure to ensure all participants could resolve three-dimensional (3D) visual cues within the VR environment. Wearing polarized glasses, participants judged four black contoured stimuli characterized by disparate elements across nine trials, identifying the circle appearing forward in the plane of reference (Garnham and Sloper, 2006). Participants were discontinued after two consecutive incorrect answers. All participants (*N* = 96) exhibited stereoacuity within the normal range (i.e., acuity better than 30 s of disparity; Bell et al., 2013) and could proceed with the experimental task. Previous research has validated the use of the Titmus test in the assessment of coarse stereoacuity (Fawcett and Birch, 2003).

##### 3.1.2.2. Apparatus

The apparatus was identical to Experiment 1. However, in Experiment 2, participants were testing individually in a quiet room, not a classroom setting. Additionally, in Experiment 2, questionnaire measures were administered online *via* Qualtrics. For the additional measure of BMI in Experiment 2, weighing scales and a tape measure were used to calculate participants’ height and weight to calculate BMI. BMI is a factor known to impact on body size judgements (Cornelissen et al., 2015; Thaler et al., 2018) and was used as a control, consistent with previous research (Dondzilo et al., 2016, 2017; Alexi et al., 2019b).

##### 3.1.2.3. Stimuli

The synthetic female body stimuli, Ebbinghaus Illusion for Bodies configuration, and bodyline task were identical to Experiment 1. Experiment 2 additionally included several questionnaire measures as detailed below.

#### 3.1.3. Measures

##### 3.1.3.1. Eating disorder examination-questionnaire 6.0

The EDE-Q (Fairburn and Beglin, 1994) is a well-validated self-report measure used in the assessment and diagnosis of eating disorder symptomatology (Peterson et al., 2007; Berg et al., 2012). The EDE-Q consists of 28 items with reference to the preceding 28 days. Twenty-two of the EDE-Q items examine the attitudinal components of eating disorder symptomatology. Participants responded to each item (e.g., “Have you had a definite fear that you might gain weight?”) using a 7-point Likert scale (0 = complete absence of feature, to 6 = acute presentation of feature). Subscale scores were averaged to produce a global EDE-Q score ranging from 0 to 6 (Mond et al., 2006). Higher scores indicated greater ED symptomatology. The remaining six items measured the frequency of one’s engagement in binge eating behaviors and were omitted from the computation of global EDE-Q scores in this study. Participant’s EDE-Q scores were used as a screening exercise to recruit eligible participants, which will be discussed in the Procedure. The Cronbach’s alpha for the 22-item global EDE-Q score in our sample was α = 0.99.

##### 3.1.3.2. Autism-spectrum quotient

The AQ (Baron-Cohen et al., 2001) is a 50-item self-report measure designed to quantify traits and behaviors associated with autism among adults within the general population. Given the considerable association between autism and eating disorder symptomatology previously discussed, we measured AQ to control for its contribution within our findings. The original item-level scoring format of the AQ coded responses dichotomously (i.e., into “agreement” or “disagreement”). Responses that endorsed the autistic trait each score one point, regardless of the strength of endorsement, while responses that do not endorse the trait score zero, also regardless of endorsement strength. Therefore, total AQ scores traditionally could range from 0 to 50. Recently, researchers have begun adopting a 1–4 Likert scoring strategy to increase scale discriminability. Such scoring strategy results in the total scale AQ scores ranging from 50 to 200. For this study, we adopted the 1–4 Likert scoring format for the AQ. Participants were required to respond to each item (e.g., “I find social situations easy”) using a 4-point Likert scale ranging from “definitely agree” (1) to “definitely disagree” (4). Higher scores indicated higher levels of autistic-like traits (Ruzich et al., 2015). The Cronbach’s alpha for the total AQ score within this sample was α = 0.83.

#### 3.1.4. Procedure

The procedure of Experiment 2 was identical to Experiment 1, except as described below. Firstly, participants voluntarily completed the EDE-Q as a screening measure prior to recruitment. Only global EDE-Q scores from female participants aged 17—24 years old were considered. In accordance with previous research comparing high and low symptomatology groups (Jansen et al., 2005; Dickter et al., 2018), global EDE-Q scores were rank ordered. Two groups were established based on the highest and lowest third of global EDE-Q scores. Only participants who were assigned to either of these groups were recruited to participate in this experiment. In total, 43 participants were recruited from the group of potential participants with high EDE-Q scores (*M* = 4.40; *SD* = 0.69) and 53 participants were recruited form the group of potential participants with low EDE-Q scores (*M* = 0.37; *SD* = 0.25). All eligible participants were contacted to participate in this study. Participants gave informed consent and completed the Titmus stereoacuity test prior to commencing the bodyline experiment.

To calculate an Ebbinghaus Illusion for Bodies’ magnitude (i.e., integration strength) for *each* participant, in Experiment 2 participants completed both small and large distractor conditions (i.e., now a within subject factor). Participants were randomly assigned to begin in one of two conditions. This was counterbalanced across all participants, with the alternative condition completed next. Following this task, participants then completed questionnaires *via* Qualtrics. Finally, each participant’s height and weight were recorded to calculate body mass index (BMI).

### 3.2. Results

#### 3.2.1. Data screening

To ensure all participants discriminated between the seven body size categories within the bodyline task, a linear regression analysis was conducted on the mean body size judgements for both Small Distractor and Large Distractor conditions separately, across body sizes 1–7. Denoting a minimum level of performance, slope values significantly greater than zero indicated that participants perceived size differences between the seven body size categories. All participants (*N* = 96) obtained a significant slope value and were all retained for further analysis.

Data were assessed for outliers. Variables were examined according to the outlier criterion of three standard deviations above or below the mean (Howell et al., 1998; Alexi et al., 2019b). All outliers that were identified in the questionnaire data, performance data, and for BMI were subsequently Winsorized (Reifman and Keyton, 2010). Next, normality was assessed using the criterion of skew <|2.00|and kurtosis <|7.00|(Curran et al., 1996; Gignac, 2019). All variables achieved sufficient normality, with skew and kurtosis values falling within accepted limits.

#### 3.2.2. Assessment of trait differences between groups

Descriptive statistics corresponding to the obtained measures are displayed in Table 2. To assess the differences between the high and low EDE-Q groups across a number of baseline dimensions, a series of independent samples *t*-tests were conducted. There was no significant difference between the EDE-Q groups in age. As expected, there was a large significant difference between the two groups on EDE-Q global score, with the high EDE-Q obtaining a significantly higher score than the low EDE-Q group. These differences are similar to those reported in other literature [as shown in (Berrisford-Thompson et al., 2021)]. Additionally, as expected, there were large significant differences in BMI, with a higher BMI for the high EDE-Q group (Berrisford-Thompson et al., 2021). Additionally, there was a significant difference in AQ total scores, with the high EDE-Q group scoring significantly higher on the AQ total, relative to the low EDE-Q group.

**Table 2:** Mean (SD) values and obtained t-test results for both high and low EDE-Q groups (*N* = 96).

Variables	High EDE-Q Group (n = 43)	Low EDE-Q Group (n = 53)	*t*	df	*p*	*d (Δ)*	95% CI
Age	19.40 (1.24)	18.74 (1.49)	−1.20	94	0.232	0.27	−0.90 0.22
BMI	23.61 (4.77)	19.80 (2.24)	5.19	94	<0.001*	0.80	2.37 5.30
EDE-Q Global	4.40 (0.69)	0.37 (0.25)	39.46	94	<0.001*	5.85	3.83 4.24
AQ Total	114.93 (11.41)	107.85 (14.38)	2.63	94	0.010*	0.62	1.73 12.43

BMI, body mass index (kg/m^2^); EDE-Q, eating disorder examination questionnaire; AQ, autism spectrum quotient. d (Δ) symbolises Glass’s delta. 95% CI = 95% confidence intervals of the difference.

* *p* values denote statistical significance.

#### 3.2.3. Spatial integration of the crowd by eating disorder symptomatology

To examine differences in spatial integration and body size judgements among individuals varying in ED symptomatology, a 2 (between-subjects variable: high vs. low ED symptomatology) × 7 (within-subjects variable: body size categories) × 2 (within-subjects variable: Small Distractor vs. Large Distractor size condition) mixed-design ANOVA was conducted. The dependent variable was the body size judgement of the central test body given on each trial which was measured using the bodyline VAS. The variables of BMI and autistic traits (AQ total) were entered as covariates in the analysis to statistically control for the influence of BMI and autistic traits upon body size judgements. All statistical assumptions were examined. The assumption of sphericity was tested using Mauchly’s Test of Sphericity. This assumption was violated for body size category and the interaction between body size category and distractor size. As such, the conservative Greenhouse–Geisser adjustment was used (Gignac, 2019). All other statistical assumptions were satisfied.

As expected, participants were able to discriminate between the seven body size categories, judging the central body to be larger as the body size category increased. This was supported by a large significant within-subjects main effect of body size category, *F*(2.26, 208.17) = 29.41, *p* < 0.001, partial *η*^2^ = 0.242.

To determine whether there is an Ebbinghaus Illusion for Bodies in VR, we looked at the difference in body size judgements when the central body is surrounded by the Small Distractors or Large Distractors. We found a small significant main effect of distractor size, *F*(1, 92) = 4.36, *p* = 0.040, partial η^2^ = 0.045. That is, participants on average are judging the central body to be *larger* when surrounded by Small Distractors and judged to be *smaller* when surrounded by Large Distractors. Therefore, an Ebbinghaus Illusion for Bodies was observed, similar to Experiment 1, even while controlling for additional variables of AQ and BMI.

**Figure 5:**
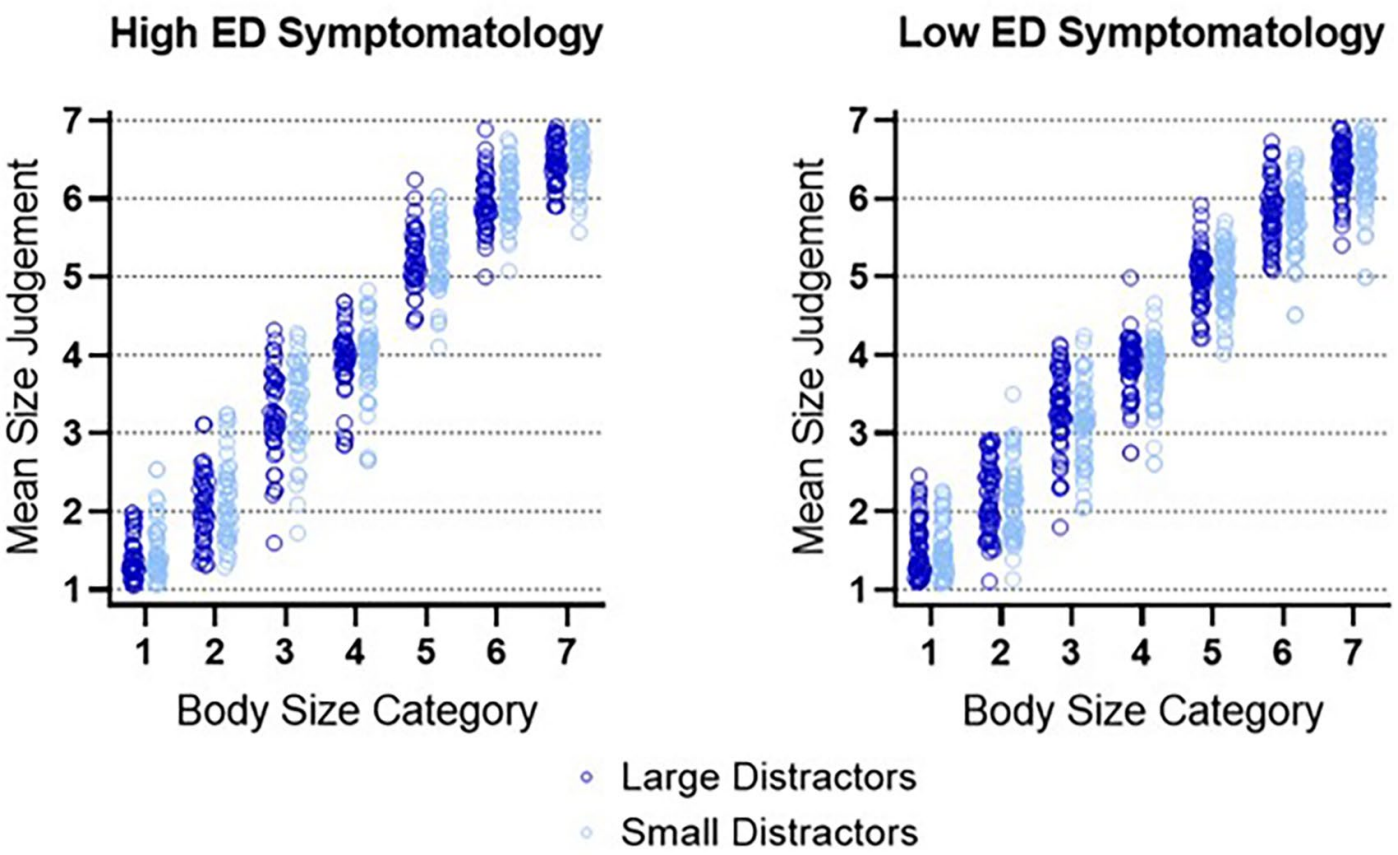
Mean individual ratings for each body size category for all participants. Created using GraphPad Prism.

Having established that our results demonstrate an Ebbinghaus Illusion for Bodies in VR, we looked at whether the high and low eating disorder symptomatology groups differed in their body size judgements. We found a small significant main effect of EDE-Q group, *F*(1, 92) = 5.66, *p* = 0.019, partial *η*^2^ = 0.058. This showed that individuals in the high EDE-Q group were providing larger mean body size judgements relative to the low EDE-Q group, as shown in Figure 5.

#### 3.2.4. Does the Ebbinghaus illusion for bodies vary across body size category?

Having established an Ebbinghaus Illusion for Bodies in VR, we next tested whether the illusion varies across body size category, akin to Experiment 1. In Experiment 2, we were able to control for BMI and AQ, and no longer find a significant interaction between distractor size and body size category, *F*(2.06, 243.16) = 2.11, *p* = 0.109, partial *η*^2^ = 0.022.

#### 3.2.5. Are individuals high in eating disorder symptomatology providing larger body size judgements, relative to individuals low in eating disorder symptomatology?

The main effect of the EDE-Q group must be interpreted with caution due to a small significant interaction effect between body size category and EDE-Q group, *F*(2.26, 208.17) = 3.57, *p* = 0.025, partial *η*^2^ = 0.037. This shows that there was variation in participant’s body size judgements across the seven body size categories as a function of the participant’s EDE-Q group.

To formalize that interpretation, we used Bonferroni’s post-hoc analyses, corrected for multiple comparisons. To do this, we collapsed each body size category across distractor condition to obtain a mean rating for each body size category. Then we compared each body size category rating across high and low EDE-Q groups. It was found that individuals high in eating disorder symptomatology were providing significantly larger judgements to body sizes category 5 and 6 relative to individuals low in eating disorder symptomatology, with *t*(94) = 3.15, *p* = 0.014, *d* = 0.64 and *t*(94) = 2.72, *p* = 0.049, *d* = 0.56, respectively. No other body size category comparisons were significant.

#### 3.2.6. Do individuals high in eating disorder symptomatology exhibit a smaller Ebbinghaus illusion, relative to individuals low in eating disorder symptomatology?

We hypothesized that individuals high in eating disorder symptomatology would exhibit a *smaller* Ebbinghaus Illusion for Bodies, relative to individuals low in eating disorder symptomatology. However, there was no significant interaction between distractor size and eating disorder symptomatology group, *F*(1, 92) = 0.52, *p* = 0.473, partial *η^2^* = 0.006. Thus, we did not find any evidence of the Ebbinghaus Illusion varying based on eating disorder symptomatology. Both groups exhibit integration and therefore our hypothesis regarding differences in spatial integration between groups was not supported.

### 3.3. Discussion

The results of Experiment 2 again showed that participants demonstrated an Ebbinghaus Illusion for Bodies in VR, even when controlling for AQ and BMI. In addition, individuals high in eating disorder symptomatology provided significantly larger body size judgements to larger bodies (Sizes 5 and 6), relative to individuals low in eating disorder symptomatology. Importantly, we did *not* find any evidence of the Ebbinghaus Illusion for Bodies varying based on eating disorder symptomatology group.

## 4. General discussion

The current study aimed to develop a novel measure of spatial integration of body size – an Ebbinghaus Illusion for Bodies in VR. Specifically, we aimed to investigate if and how the perceived size of a female body is influenced by the presence of other bodies within the scene. We achieved this aim. For Experiment 1, it was hypothesized that there would be an Ebbinghaus Illusion for Bodies, that is, participants would judge the central body to be larger when surrounded by a crowd of very thin bodies (the Small Distractors), and vice versa. As predicted, we observed an Ebbinghaus Illusion for Bodies in VR, indicating that spatial integration of crowd information can lead to biased body size perception. It was found that the central body was perceived to be *larger* when surrounded by Small Distractors and perceived to be *smaller* when surrounded by Large Distractors, thus supporting our hypothesis. Additionally, the effect of a crowd on body size judgements was not consistent, with variation in the magnitude of the Ebbinghaus Illusion for Bodies across body size category, as expected from work on the traditional Ebbinghaus Illusion (Roberts et al., 2005).

In Experiment 2, we investigated whether the Ebbinghaus Illusion for Bodies varied amongst individuals differing in eating disorder symptomatology. It was hypothesized that individuals high in eating disorder symptomatology would exhibit a *smaller* Ebbinghaus Illusion for Bodies, relative to individuals low in eating disorder symptomatology. We replicated the finding that participants demonstrated an Ebbinghaus Illusion for Bodies. However, the illusion did not vary based on eating disorder symptomatology. Therefore, our hypothesis that higher eating disorder symptomatology would exhibit a smaller Ebbinghaus Illusion for Bodies was not supported.

Our findings in both Experiment 1 and Experiment 2 demonstrated an Ebbinghaus Illusion for Bodies in VR, which indicated that spatial integration of crowd information can lead to biased body size perception. Furthermore, even when controlling for the appropriate variables including BMI and traits associated with autism (measured by AQ) in Experiment 2, the Ebbinghaus Illusion for Bodies remained significant. Our results provide evidence of a further mechanism leading to body size misperceptions: surrounding spatial information (external to the target body) is integrated and can influence one’s judgement of body size.

Both experiments builds upon the work of Bateson et al. (2014), revealing the importance of looking at a range of body sizes. As revealed in Experiment 1, there was variation in the magnitude of the Ebbinghaus Illusion for Bodies, across body size category. That is, the integration of spatial information influences one’s judgements of body sizes, however this does not occur equally for all target bodies. In Experiment 1, this was reflected in a non-significant Ebbinghaus Illusion for the Size 1 body and for largest body sizes (5–7). For the larger bodies, this result was somewhat unsurprising given that previous literature has shown that body size judgement accuracy is related to body size. As shown by Cornelissen et al. (2016), as bodies become overweight and obese, it becomes increasingly difficult for individuals to judge their body weight or to detect an increase in size (known as Weber’s law, where the “just noticeable difference” between two stimuli becomes larger as the magnitude of the judged object increases). By systematically manipulating target and distractors body sizes, our results characterize how the size relationship between target and distractors influences the magnitude of this body size illusion. It seems that the Ebbinghaus Illusion for Bodies is the most noticeable when the target is a thin to average size female body, distinct in size from the surrounding crowd.

Despite the substantial amount of research regarding integration difficulties and weak central coherence in eating disorder populations (Lopez et al., 2008a,b,c; Blumberg et al., 2014; Lang et al., 2014a, 2021; Keegan et al., 2021), the illusion did not significantly differ between individuals high and low in eating disorder symptomatology as hypothesized. There could be several plausible reasons for this result. First, eating disorder symptomatology is a continuum ranging from normal eating behaviors, through to elevated eating disorder symptomatology, and clinical eating disorders (Shisslak et al., 1995). Therefore, while Experiment 2 used a representative sample of the extreme (e.g., high, and low) eating disorder symptomatology groups, this still may limit the degree to which these current findings can be generalized to a clinical eating disorder population. In saying this, the high eating disorder symptomatology group demonstrated higher levels of eating disorder symptomatology relative to previous research investigating these higher-risk groups (Jansen et al., 2005; Berrisford-Thompson et al., 2021) and even samples containing current and recovered eating disorder patients (Engel et al., 2022). Regardless, given that our high eating disorder symptomatology group was not a clinical sample, it may be possible that this group did not display a high enough severity of eating disorder symptoms. Furthermore, the EDE-Q used in this study provides an overall measure of eating disorder symptomatology (e.g., eating concern, restraint, weight concern, and shape concern) but is unable to discriminate between the eating disorder diagnoses. Given the higher BMI found in this high eating disorder symptomatology group, it may be possible that many of our participants in the high group had symptoms in line with bulimia nervosa, binge eating disorder, and other specified feeding and eating disorders rather than anorexia nervosa. Future research could extend these questions and methodology to those diagnosed with a clinical eating disorder to compare to the general population. In addition, this design may still be relevant to other clinical populations. As previous discussed, research has found that spatial integration difficulties, such as weak central coherence, are proposed to underlie core symptomatology of ASD (Frith, 1989). Therefore, this test of integration may be of interest to explore with other clinical populations displaying spatial integration difficulties, such as individuals differing in levels of traits associated with autism, and whether these difficulties extend to influencing upon their body size judgements.

While we found an Ebbinghaus Illusion for Bodies in VR in both experiments, it was characterized by a small effect size. It is possible that the size of our Ebbinghaus Illusion for Bodies was too small to usefully demonstrate reductions in the size of the illusion/spatial integration according to eating disorder symptomatology. It is possible a modification of the task would increase the effect size for the Ebbinghaus Illusion for Bodies in VR. Roberts et al. (2005) identified several factors that impact on the magnitude of the Ebbinghaus Illusion. One factor is the spacing between inducers and the central target, which may be compressed or expanded, depending on the size of the elements. Spacing the targets and distractors was challenging in our design, particularly in the Large Distractor condition, where the larger bodies created occlusions between distractors, potentially reducing their effectiveness (see Figure 1B). In addition, it was also possible for the inducers to occlude the central target body itself. By comparison, target occlusions were greatly reduced if not entirely absent for smaller body sizes. These occlusion effects created by the larger body sizes may also have contributed to the small or absent illusion for large to extreme (Sizes 5–7) body sizes in Experiment 1. Future research could adjust the spacing, size, posture and positioning of the surrounding inducer bodies.

It also important to acknowledge that there may be potential ceiling and floor effects reducing the magnitude of the Ebbinghaus Illusion for Bodies. As previously discussed, Experiment 1 showed a non-significant Ebbinghaus Illusion for the smallest (Size 1) and largest (Size 7) bodies. Average judgements of a Size 1 body are less than 1.5, close to the end of the VAS. When participants are required to judge a Size 1 body surrounded by Size 7 distractors, being near the end of the scale already may inhibit the judgement and becoming biased toward an even smaller body size (floor effect). The reverse, or ceiling effects may also impact the size of the illusion for Size 7 judgements. Future studies could also consider running this experiment using only target body sizes 2–6. By doing so, we would be able to ensure that the distractors will always be bigger or smaller than the target stimuli.

Overall, it seems useful to note that our study does demonstrate that individuals high in eating disorder symptomatology *are* integrating spatial information and do not vary in the magnitude of this integration relative to individuals low in eating disorder symptomatology. The lack of significant difference between our high and low groups suggests that that spatial integration difficulties may not represent in tasks involving integration across a crowd when making body size judgements. That is, when the integration involves multiple discrete objects in a scene. This test of integration may also be of interest to explore *what types* of information from the surrounding inducers when making body size judgements, including the integration of local body features (e.g., hip-to-waist ratios, stomach circumference, etc.) or more holistic features (e.g., the body as a whole). Although this is not to say that these spatial integration difficulties would not emerge for a clinical eating disorder sample, these findings further help researchers understand when spatial integration difficulties emerge (or not) for individuals high in eating disorder symptomatology.

It seems plausible that the integration difficulties reported for individuals high in eating disorder symptomatology are specific to the integration of information within a single body, rather than across a scene. The importance of the single body is further reinforced by our results in Experiment 2, where we did observe differences between those high and low in eating disorder symptomatology, on single target body size judgements. This shows that how a single *target* body was judged differentiates between the high and low eating disorder symptomatology groups, rather than differences in spatial integration across the crowd of bodies. This leaves open the possibility that integration difficulties reported in previous literature are restricted to incidences involving a single target body. Therefore, our findings further support the use of single bodies in investigating integration difficulties within the context of eating disorder symptomatology.

The findings of this study provide an important extension to our limited understanding to how features *external* to a single target body can influence its perceived size. As previously discussed, to our knowledge, only Bateson et al. (2014, p. 195) has reported the impact that other bodies can have on size judgements. Our study further extends upon the findings of Bateson et al. (2014) in multiple ways. First and foremost, our results broaden understanding of bystander influence by systematically investigating and mapping how this size illusion varies as a function of target body size and of the target and distractor size difference. Next, we implemented this effect in a 3D setting, a novel extension to Bateson et al. (2014). Furthermore, we directly measured changes in body size judgements, rather than a related but indirect construct measuring change in the probability of being assigned the label of larger versus smaller in simultaneous stimuli presentations, as in Bateson et al. (2014).

Finally, we studied bystander influence using 3D bodies within a VR environment. This is a potentially important consideration given research showing that 3D cues can enhance and/or alter sensitivity to objects and object recognition (Bennett and Vuong, 2006; Burke et al., 2007; Bell et al., 2013; Caziot and Backus, 2015). In addition, Turnbull et al. (2022) found that 3D cues influence body size judgements by providing viewers with increased stimulus certainty (e.g., addition of volumetric information of lower stomach and bust regions). Furthermore, from an ecological perspective, the bodies that we often see in real life are surrounded by other people. By representing the crowd of bodies in 3D, we were able to create a design where the crowd of bodies could exist without occluding each other.

Our research also has limitations that should be acknowledged. First, using computer-generated (CG) stimuli prompts one limitation that the precise physical weight and thus BMI of our CG stimuli were unknown. Therefore, we cannot provide an exact estimate of veridical performance. Additionally, the use of CG stimuli over the use of real body images may also pose other limitations. Alexi et al. (2019a) have shown that using CG stimuli reduces discriminability between extreme body categories. This has been hypothesized to be due to the impoverished representation of textural elements in extreme CG imagery, such as the lack of cellulite or hollowed skin surfaces, which may be used as markers of body weight (Alexi et al., 2019a). However, it should be noted that research by Cornelissen et al. (2016) and Tovée et al. (2012) found comparable findings between real body images and CG stimuli. Additionally, the use of CG stimuli may have its advantages in allowing researchers to systematically control for body characteristics, such as weight and size, and allow for consistency between body size categories (Moussally et al., 2017). This study ensured that on each trial, each body varied in appearance, clothing and overall lighting. Furthermore, in our study, our CG stimuli have the advantage of including stereoscopic (e.g., three-dimensional) cues, which provide further realism and ecological validity, relative to 2D CG stimuli used in previous studies (Turnbull et al., 2022).

We also acknowledge that our study presented female body stimuli, but not male body stimuli. This is important to consider given that judgements of male and female bodies may differ. For context, research has shown that judgements of male body size are mediated by both body fat *and* muscularity (Adams et al., 2005; Jones and Crawford, 2005; Tylka, 2011). Furthermore, adiposity and muscularity are shown to be two independent dimensions considered in perceptions of body size and composition (McCreary et al., 2006; Coetzee et al., 2010; Sturman et al., 2017), however this distinction has not been captured in our stimulus continuum. Since bodies also vary in muscle mass, particularly for men, future studies may explore whether the Ebbinghaus Illusion for Bodies occurs using male body stimuli. Furthermore, while the focus of this study has been on a female demographic shown to be most at risk of development of an eating disorder (Hudson et al., 2007; Stice et al., 2013; Rohde et al., 2017), it is important to acknowledge that peak onset for eating disorders in men is at aged 18—20 years old (Allen et al., 2013; Raevuori et al., 2014). Given the increasing rates of body dissatisfaction and eating disorders in young men (Dakanalis et al., 2016), it would be important to re-create Experiment 2 using male body stimuli *and* male participants varying in eating disorder symptomatology. This would help us understand whether the Ebbinghaus Illusion for Bodies differs based on the gender of the stimuli and eating disorder symptomatology of the sample used. This would further contribute to a robust understanding of spatial integration and weak central coherence within males high in eating disorder symptomatology.

In summary, using a novel design, we wanted to understand the factors that can impact upon body size judgements. While previous research has opted to look at the judgement of a *single*, *isolated* test body, this study demonstrates that spatial integration of a crowd of surrounding bodies can influence body size judgements. In the general population, it was found that the integration of the Large Distractors resulted in the target body being perceived as smaller and the integration of the Small Distractors result in the target being perceived as larger. Critically, our results revealed that those high and low in eating disorder symptomatology demonstrated equivalent integration. Using VR technology to simulate a crowd and increase ecological validity, this study provides a novel explanation of why we make errors in body size judgements. Overall, the findings add to the growing body of literature demonstrating the malleability of body size judgements and provide further support on the design choices of researchers looking to examine body size and shape judgement errors.

## Data availability statement

The raw data supporting the conclusions of this article will be made available by the authors, without undue reservation.

## Ethics statement

The studies involving human participants were reviewed and approved by The University of Western Australia Human Research Ethics Committee. Approval #: 2021/ET000004. Written informed consent from the participants’ legal guardian/next of kin was not required to participate in this study in accordance with the national legislation and the institutional requirements.

## Author contributions

GT, SL, BK, and JB designed the study. YL provided technical assistance with the virtual reality, stimuli creation, and software. Testing and data collection were performed by GT, SL, BK, JA, GM, and JB. All authors contributed to the article and approved the submitted version.

## Conflict of interest

The authors declare that the research was conducted in the absence of any commercial or financial relationships that could be construed as a potential conflict of interest.

## Publisher’s note

All claims expressed in this article are solely those of the authors and do not necessarily represent those of their affiliated organizations, or those of the publisher, the editors and the reviewers. Any product that may be evaluated in this article, or claim that may be made by its manufacturer, is not guaranteed or endorsed by the publisher.

## Footnotes

^1^As a preliminary analysis, gender was included as an independent variable. There was no significant effect of gender on body size judgements. Given that gender was not the primary focus of this study, no further analyses including gender were conducted.
